# Designing bike networks using the concept of network clusters

**DOI:** 10.1007/s41109-018-0069-0

**Published:** 2018-06-18

**Authors:** Meisam Akbarzadeh, Syed Sina Mohri, Ehsan Yazdian

**Affiliations:** 1 Department of Transportation Engineering, Isfahan University of Technology, Isfahan, Iran; 2 Department of Electrical and Computer Engineering, Isfahan University of Technology, Isfahan, Iran

**Keywords:** Graph, Cluster, Bike network design, Smart card data

## Abstract

In this paper, a novel method is proposed for designing a bike network in urban areas. Based on the number of taxi trips within an urban area, a weighted network is abstracted. In this network, nodes are the origins and destinations of taxi trips and the number of trips among them is abstracted as link weights. Data is extracted from the Taxi smart card system of a real city. Then, Communities i.e. clusters of this network are detected using a modularity maximization method. Each community contains the nodes with highest number of trips within the cluster and lowest number of trips with other clusters. Within each community, the nodes close enough to each other for being traveled by bicycle are detected as key points and some non-dominated bike network connecting these nodes are enumerated using a bi-objective optimization model. The total travel cost (distance or time) on the network and the path length are considered as objectives. The method is applied to Isfahan city in Iran and a total of seven regions with some non-dominated bike networks are proposed.

## Introduction

Promotion of non-motorized transportation is a step toward sustainable urban development. The benefits of travel by cycling and walking include increased physical health, decreased dependence on fossil fuel combustion, decreased production of environmental pollutants, efficient use of capacity of urban passages, and provision of more equitable conditions due to lack of dependence on citizens’ economic and car ownership status. Promotion of non-motorized forms of transportation requires requires proper infrastructure and service. In the case of cycling, the presence of bike-lanes with suitable safety, geometric design and pavement can have a significant impact on citizens’ willingness to use bicycles for short and medium range travels. Common methods of identification of suitable routes for construction of a bike network are based on two principles: i) determination of urban passages suited for allocation of necessary width to bike-lanes, and ii) identification of origins and destinations of short and medium range travels. These origins and destinations can be identified by direct statistical surveys (through observation and questionnaire) or indirect use of past data (the outputs of comprehensive urban transportation plans that have been developed based on direct surveys).

Statistical surveys are based on rigorous scientific principles; however, the presence of inevitable errors (e.g. sampling error), the high cost of collecting adequate sampling, and the difficulty of securing the effective cooperation of respondents make these surveys a challenging phase of transportation studies. The widespread use of intelligent transportation systems however allows researchers to extract useful information about citizens’ travel behavior without the need for any direct engagement. Recently, the presence of automated vehicle location systems, automatic transit fare collection systems, speed cameras and license plate scanners provide unprecedented access to raw data necessary for the study of traffic behaviors.

The method proposed in this paper is based on data pertaining to taxi trips and does not therefore require any direct survey. In this method, origins and destinations of short taxi trips are abstracted as vertices of a graph. Short trips are those within the feasible distance traversed by bike which is assumed 4 km in this study. If a trip is made between two vertices, they become connected by an arc. The number of travels between two points is modeled as the weight of the arc connecting the corresponding vertices. Modeling the travel patterns as a graph paved the way for using the concept of community i.e. clusters to identify the points with more significant travel connections. On this basis, after detecting the graph communities, the point with highest rates of short-range trips in each community were identified, and then the best networks connecting these points was attained based on a bi-objective mathematical model. The first objective of the model minimizes the total travel cost (distance or time) on the network as a users’ objective. While, the second objective minimizes the total network length as planners’ objective. Therefore, the model by considering a trade-off between users and planners objectives proposes some non-dominated (pareto-optimal) bike networks.

The rest of the paper is organized as follows: A review of application of graph theory in transportation networks, the usage of information of taxi positioning systems, and methods of bike network design are presented in section “Review of literature”. In section “Research method”, a methodology of identifying the non-dominated bike networks in a city is proposed based on integrating a community detection method and a bi-objective optimization problem. Section “Data and results” is devoted to analyzing the results of applying the presented method on a real case study of Isfahan network.

## Review of literature

The literature review in this study follows of three streams, methods of analysis the complex networks and their applications, the usage of extracted information of taxi positioning system on urban planning and the method of designing bike networks.

### Graph theory and complex networks

Graph theory and complex networks have found many applications in air, sea, rail and land (highways and public) transportation networks. Previous studies in this field are mainly focused on identification of network’s functional communities, vulnerably (Hu and Zhu 2009; Li and Cai 2007; Mohmand and Wang 2014), reliability (Duan and Lu 2014; Qian et al. 2012), evolution pattern (Jia et al. 2014; Roth et al. 2012), and comparative studies on different networks through performance measurements (Leng et al. 2014; Von Ferber et al. 2009; Xu et al. 2007).

**Fig. 1:**
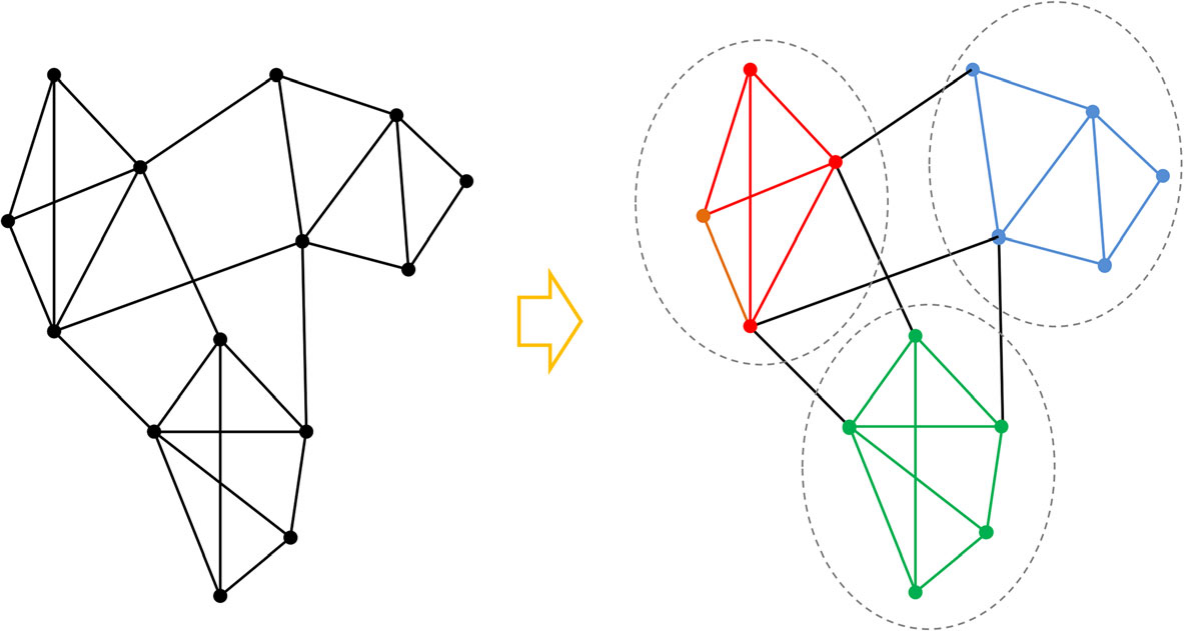
Communities of a sample network

One of the applications of network-based approach is the identification of potential community of a network. In a graph, community also known as cluster is a subgraph whose vertices have a high degree of inter-connection and relatively low connection with vertices outside that subgraph. Figure 1 shows an example of communities in a simple network.

In large and complex networks, communities cannot be detected by shear intuition; but literature has provided several methods for this purpose. These methods can be grouped into two categories: division methods, and aggregation methods (Clauset et al. 2004; Girvan and Newman 2002; Newman 2006; Newman and Girvan 2004; Pons and Latapy 2005; Radicchi et al. 2004; Wu and Huberman 2004). Division methods assume the entire network as a large community and then select the vertices most suited for isolation. These methods divide the network to its communities and continue this process until the generated communities exhibit the desired quality, i.e. when vertices of each community have a high degree of inter-connection and relatively low connection with vertices of other communities. Aggregation methods first assume each vertex as a minuscule community, and then determine the vertex most suited for formation of a larger community (containing two vertices). These methods aggregate the vertices to form the most suitable communities, and then examine the addition of remaining vertices to existing communities, and repeat this process until the generated communities exhibit the above-mentioned quality.

### The usage of information of taxi positioning systems

The information obtained from automated positioning system of taxis have been used in numerous transportation and urban planning studies. Previous studies in this regard have mostly focused on developing and updating street maps (Cao and Krumm 2009; Lou et al. 2009), developing transportation routes and services based on frequent patterns of taxi trips (Chen et al. 2013; Wei et al. 2012; Ziebart et al. 2008), predicting the time and volume of traffic in city streets and identifying the points with frequent traffic jams (Castro et al. 2012; Gao et al. 2013; Liu et al. 2010b; Wang et al. 2009; Zhu et al. 2011), classifying the land use by analyzing the information regarding the arrival and departure of passengers over space and time dimensions (Pan et al. 2013; Yuan et al. 2012), recommending optimal routes during rush hours based on route selected by taxis (Liu et al. 2010a; Yuan et al. 2010), predicting the dynamic patterns of travel distribution by analyzing factors such as time, location of taxis, and weather conditions (Chang et al. 2009; Yue et al. 2012), identifying the unknown connections in the network of intra-urban travel (Zheng et al. 2011), and identifying the nearest source of passengers for vacant and roaming taxis (Veloso et al. 2011; Yuan et al. 2011).

This study, by applying the clustering method on the information of taxi trips, where are gathered by a digital payment service, identifies the potential locations (key bike nodes) of a city for setting up a bike network. Considering these potential locations as some small networks instead of the whole city network, reduces the size of problem with preserving the quality of results for designing a bike network facility.

### Bike network design

Several studies represents that the countries and cities with a high cycling demand in Western Europe and North America have large networks of separate bike facilities (Fraser and Lock 2011; Furth 2012; Pucher et al. 2010). In contrast with the other transportation network design, the cyclists considers a broader range of factors for selecting routes such as travel time, distance, comfort, slope, turn frequency, noise, pollution etc. (Broach et al. 2012; Winters et al. 2011). Therefore, designing the bike networks or routing the bike lanes usually is done based on some different criteria. There is a difference between routing bike lanes and bike network design. The objective of routing problem is proposing some best routes between a specific origin and destination (OD). While, the bike network design problem considers some OD pairs and presents some directed bike lanes as a bike network (Buehler and Dill 2016; Hrncir et al. 2015; Mauttone et al. 2017; Song et al. 2014).

Buehler and Dill (2016) with reviewing the literature reported the different approaches to design the cycling infrastructures such as links, nodes and network. They concluded that designing a bike network as a whole is the much remained approach for planning cycling infrastructures. The literature on the topic of bike network design is relatively scarce. Mesbah and Thompson (2011) presented a bi-level optimization model for bike network design. The upper-level simultaneously maximized the share of bike trips and its impact over car travel time due to reduction of street space. The lower-level was a traffic assignment for both bikes and cars with a user-equilibrium hypothesis (Mesbah and Thompson 2011). Duthie and Unnikrishnan (2014) proposed a single objective optimization model which aimed to decrease the total constructing costs of a bike network in a city. It was assumed that the total bike OD demand in network must be covered with the proposed network. Also, the construction costs of network was related to the links and intersections (Duthie and Unnikrishnan 2014). Mauttone et al. 2017, with considering the interest of planners and users proposed a single objective model for bike network design that minimized the distance of bike trips given by an OD matrix. The interest of planner was provided by applying a budget constraint into the model (Mauttone et al. 2017).

This study with considering both objectives of planners and users proposes a bi-objective model for bike network design. In contrast with (Mauttone et al. 2017), we consider the interest of planner as a model objective with minimizing the length of proposed network. Also, the potential OD demands for bike network are gathered by a digital payment service as a revealed preferences data. The previous studies built the OD matrix with a stated preferences data that were collected by home surveys (Duthie and Unnikrishnan 2014; Mauttone et al. 2017; Mesbah and Thompson 2011). One of the big problem of previous studies for routing bike lanes or bike network design was the the big size of the problem and disability of exact methods for solving it (Hrncir et al. 2015; Mauttone et al. 2017; Song et al. 2014). This study with identifying the key OD pairs in each cluster that have the most potential of moving to bike network decreases the size and complexity of problem.

## Research method

**Fig. 2:**
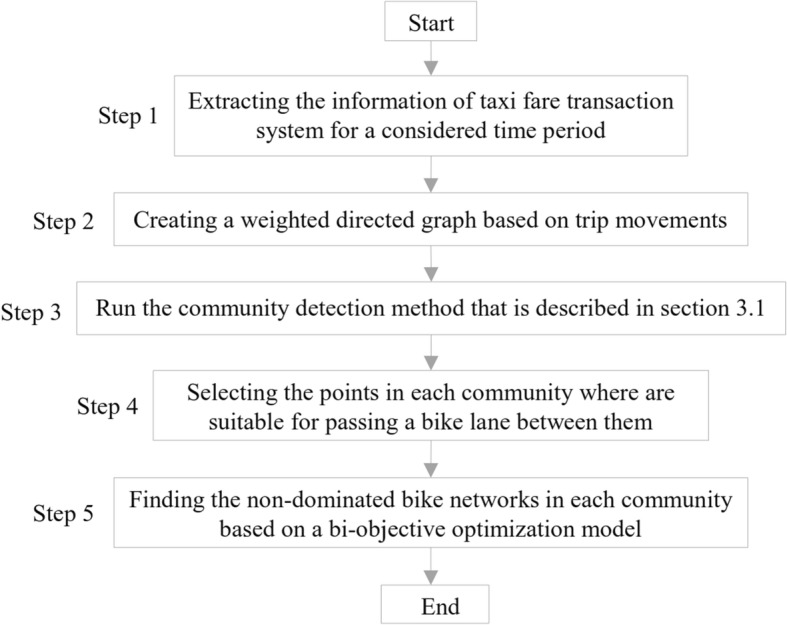
The framework of the methodology

The objective of present study is to determine the routes most suited for the development of a bike network by analyzing the matrix of taxi trips based on the data obtained from digital payment service. Figure 2 shows the methodology as a flowchart.

Steps are explained in detail in the following subsections.

*Step 1: Extract the taxi trip data for a time period*

Taxi trip data was extracted from the fare transaction system of Isfahan Taxi Organization. The database was anonymized and included the longitude and latitude of the trip origin and destination, and the boarding and alighting time of each passenger.

*Step 2: Create a weighted graph based on trip patterns*

Every trip origin and destination could potentially be considered as a node of the graph. This would yield a huge graph. Hence, trip origins and destinations close to each other were aggregated and contracted to one node. Corresponding trips of aggregated nodes were also aggregated. The method of aggregation is described in section “Data and results”.

*Step 3: Detect the communities of the graph*

Community detection was conducted by the heuristic algorithm presented by Blondel et al. (Blondel et al. 2008) applicable to undirected networks. The algorithm consists of two consecutively repeating steps. In the first step, algorithm considers each vertex of the network as a community. In the second step, algorithm identifies the two vertices with the most interaction and groups them as one community. It then replaces these two vertices with one (virtual) vertex and repeats the first step. In this algorithm, the suitability of vertices for aggregation is determined by the value of modularity. Modularity (Q) is a variable that compares the density of intra-community and inter-community connections, and as a result, its value represents the quality of the formed communities. In a weighted network, this index is defined as the following equation:

1 $$ Q=\frac{1}{2m}\sum \limits_{i,j}\left[{A}_{ij}-\frac{K_i{K}_j}{2m}\right]\updelta \left({C}_i,{C}_j\right) $$

where *A*_*ij*_ denotes the weight of the arc connecting vertex i to vertex j; *K*_*i*_ represents the total weight of all arcs connected to vertex i; *C*_*i*_is the community that includes the vertex i; δ is a binary function which is 1 when i and j are in the same community, and is 0 otherwise; and 2*m* is the total weight of all arcs in the network.

The community detection algorithm first selects an arbitrary vertex (i), separates it from its community and inserts it into the neighboring community (j), and then recalculates the resulting modularity index. It repeats this process for all vertices adjacent to vertex i, and ultimately adds the vertex i to the neighboring community with maximum positive ΔQ (difference between modularity index of target community with that of original community). The change in the modularity index (ΔQ) is calculated by eq. (2):

2 $$ \Delta  Q=\left[\frac{\sum_{in}+{K}_{i, in}}{2m}-{\left(\frac{\sum_{tot}+{K}_i}{2m}\right)}^2\right]-\left[\frac{\sum_{in}.}{2m}-{\left(\frac{\sum_{tot}.}{2m}\right)}^2-{\left(\frac{K_i}{2m}\right)}^2\right] $$

where ∑_*in*_.is the total weight of all arcs inside the community C; *K*_*i*, *in*_ is the total weight of all arcs connecting the vertex i to other vertices of the community C; and ∑_*tot*_. is the total weight of all arcs connected to the vertices of the community C.

The algorithm repeats this process for all vertices in the network and continues until ΔQ cannot be improved any further. In the second step, algorithm considers each formed community as one vertex and considers the total weight of connections between the two communities of the first step as the weight of the new arc. This leads to formation of a new network whose layout and properties depends on the output of the first step. This algorithm then repeats the entire process for the new network. This second step of algorithm continues until ΔQ cannot be further improved. This marks the end of algorithm’s first cycle (iteration) and the start of a new cycle through re-initiation of step1. These iterations continue until modularity index cannot be improved any further.

*Step 4: Select the links in each community suitable for passing bike lanes*

Suitable distance for biking was assumed to be four kilometers. Hence, in each community node pairs with distances less than 4 km were selected as potential bike lane routes. Hereafter, these nodes are called the key points.

*Step 5: Find the best bicycle route in each community*

**Fig. 3:**
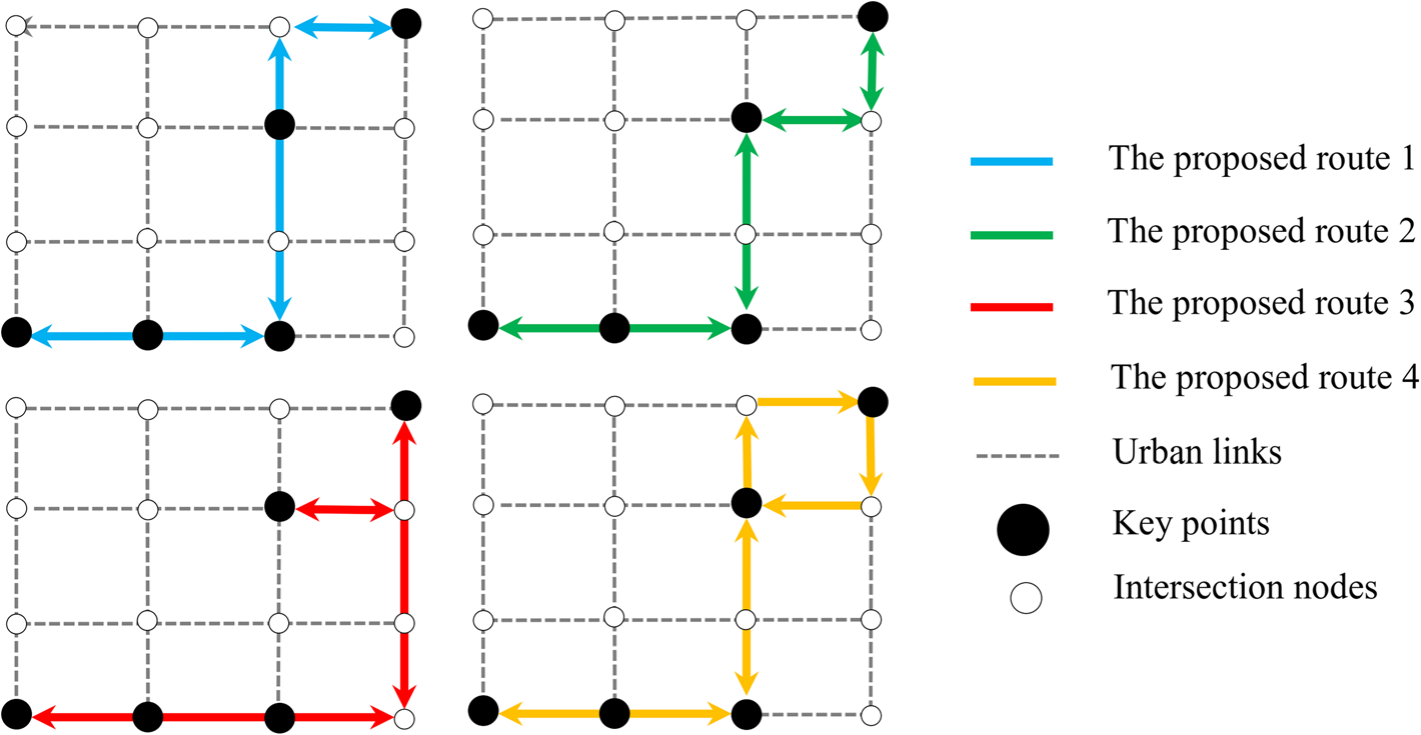
Alternative bike networks within a community: an example

Routes connecting the key points in each community were enumerated to generate the choice set for selecting the best route for constructing the bike network. An economical and desirable network should consider a trade-off among the goals of users and planners. Accordingly, we present a bi-objective optimization model for bike network design problem to generate some non-dominated solutions and facilitate the process of decision making. The mixed integer formulation of model is a variant of fixed-charge multi commodity network design problem (Magnanti and Wong 1984). The first problem objective is minimizing the total travel costs (distance) as the users’ objective. The second objective minimizes the total length of proposed directed bike network as the planners’ objective. This objective covers the economic issues for constructing the bike network and has a conflict with the first one. An example is illustrated in Fig. 3 to explain how the optimization model works. Assume a grid network in which five nodes have high values of short-length taxi trips.

Figure 3 shows four possible networks selected as the non-dominated bike networks. A non-dominated solution is a solution for which each objective could not be improved without deteriorating the other objectives. There is a large variety of classical methods for converting a multi-objective model to a single objective one and generally, none of them can be said to be superior to others (Hartikainen et al. 2012). For example, the values of objectives (*O*_1_, *O*_2_) for these four networks are as follow: (100, 20), (80, 30), (60, 35), and (50, 40).

In this study, a weighted method with normalization is used to convert the bi-objective to a single objective model. The weighted method with normalization is an extension of weighting method in which the objectives are normalized to return a value between zero and one (Grodzevich and Romanko 2006). Normalization of each objective is done by deducing the value of ideal solution of the objective formulation and dividing them by different values between the nadir and the ideal solutions of the objective function. For a bi-objective model the ideal solution for each objective is obtained by minimizing it without considering the other objective. Also, when the first objective is minimized, the value of second objective is a nadir solution for second objective and vice versa. Equation (3) shows the process of normalization for objective *i*. Equation (4) demonstrates the new weighted objective constructed from initial two objectives.

3 $$ {f}_i^{\prime }(x)=\frac{f_i(x)-{f^L}_i}{{f^N}_i-{f^L}_i} $$

4 $$ h(x)={w}_1{f}_1^{\prime }(x)+{w}_2{f}_2^{\prime }(x) $$

Where, *f*_*i*_(*x*) and ${f}_i^{\prime }(x)$ are the objective function *i* and its normalized form, respectively. *f*^*L*^_*i*_ and *f*^*N*^_*i*_ are the ideal and nadir solutions of objective function *i*, respectively. *h*(*x*) is the new weighted objective constructed from initial two objectives. *w*_1_ and *w*_2_ are the weights of the first and second objectives, respectively. Also, in this method the sum of weights must be equal to one. In this study, for extracting a set of non-dominated solutions the weight of first objective increases from zero to one by steps equal to 0.1.

Before describing the mathematical problem of bi-objective bike network design, the used sets, indices, input parameters and decision variables are described.

- Sets

**Table:** 

*N*	: The set of network nodes
*A*	: The set of network links
*D*	: The set of network demands

- Indices

**Table:** 

*s*	: The index for network demands
*i*, *j*	: The index for network nodes

- Input parameters

**Table:** 

*L*_*ij*_	: The length of link (*i, j*)
*Q*_*s*_	: The amount of OD demand flow *s*
*O*(*s*)	: The origin node of OD demand flow *s*
*D*(*s*)	: The destination node of OD demand flow *s*
*M*	: A constant positive number and it is equal to the number of OD demand flows in the network

- Decision variables

**Table:** 

${x}_{ij}^s$	: A binary decision variable, it is equal to one if link (*i, j*) be selected for routing OD demand flow *s*, otherwise it is equal to zero.
*Z*_*ij*_	: A binary decision variable, it is equal to one if link (*i, j*) be selected as a network link, otherwise it is equal to zero.

5 $$ \mathit{\operatorname{Min}}\ {O}_1=\sum \limits_{s\in D}\sum \limits_{i\in N}\sum \limits_{j\in N,\left(i,j\right)\in A}{x}_{ij}^s\times {L}_{ij}\times {Q}_s $$

6 $$ \mathit{\operatorname{Min}}\ {O}_2=\sum \limits_{i\in N}\sum \limits_{j\in N,\left(i,j\right)\in A}{Z}_{ij}\times {L}_{ij} $$

$$ St: $$

7 $$ \sum \limits_{j\in N,\left(i,j\right)\in A}{x}_{ij}^s-\sum \limits_{j\in N,\left(j,i\right)\in A}{x}_{ji}^s=1\kern2.5em \forall i\in N,\forall s\in D, and\ i=O(s) $$

8 $$ \sum \limits_{j\in N,\left(j,i\right)\in A}{x}_{ji}^s-\sum \limits_{j\in N,\left(i,j\right)\in A}{x}_{ij}^s=1\kern2.5em \forall i\in N,\forall s\in D, and\ i=D(s) $$

9 $$ \sum \limits_{j\in N,\left(i,j\right)\in A}{x}_{ij}^s-\sum \limits_{j\in N,\left(j,i\right)\in A}{x}_{ji}^s=0\kern2.5em \forall i\in N,\forall s\in D, and\ i\ne \left\{O(s),D(s)\right\} $$

10 $$ \sum \limits_{s\in D}{x}_{ij}^s\le M{Z}_{ij}\kern2.25em \forall i,j\in N, and\left(i,j\right)\in A $$

11 $$ {x}_{ij}^s\  and\ {Z}_{ij}\in \left\{0,1\right\} $$

Equation (5) is the users’ objective and minimizes the total travel distance in the network. Equation (6) is the system or planners’ objective that minimizes the total length of bike network. Equations (7), (8), and (9) are the flow conservation constraints. Equation (7) ensures that for each OD pair, a network link is departed from origin. Equation (9) expresses that for each OD pair, a link must arrive at the destination of the OD demand. Equation (9) is for intersection nodes and ensures that if a link enters an intersection node another link for leaving it must exist. Based on Eq. (10) if link (*i, j*) is selected for transferring the OD flows, this link must be constructed in the network. Finally, Eq. (11) shows the nature of the decision variables (binary variable).

## Data and results

Taxi trip data of Isfahan, Iran was used for implementing the model. Accordingly, travel information within the period of 26 May 2014 to 30 May 2014 of all the taxis equipped with the smart card system were obtained. The database contained the coordinates of trip origins and destinations, trip duration, and actual distances traveled. The database contained nearly fifty-three thousand trips.

**Fig. 4:**
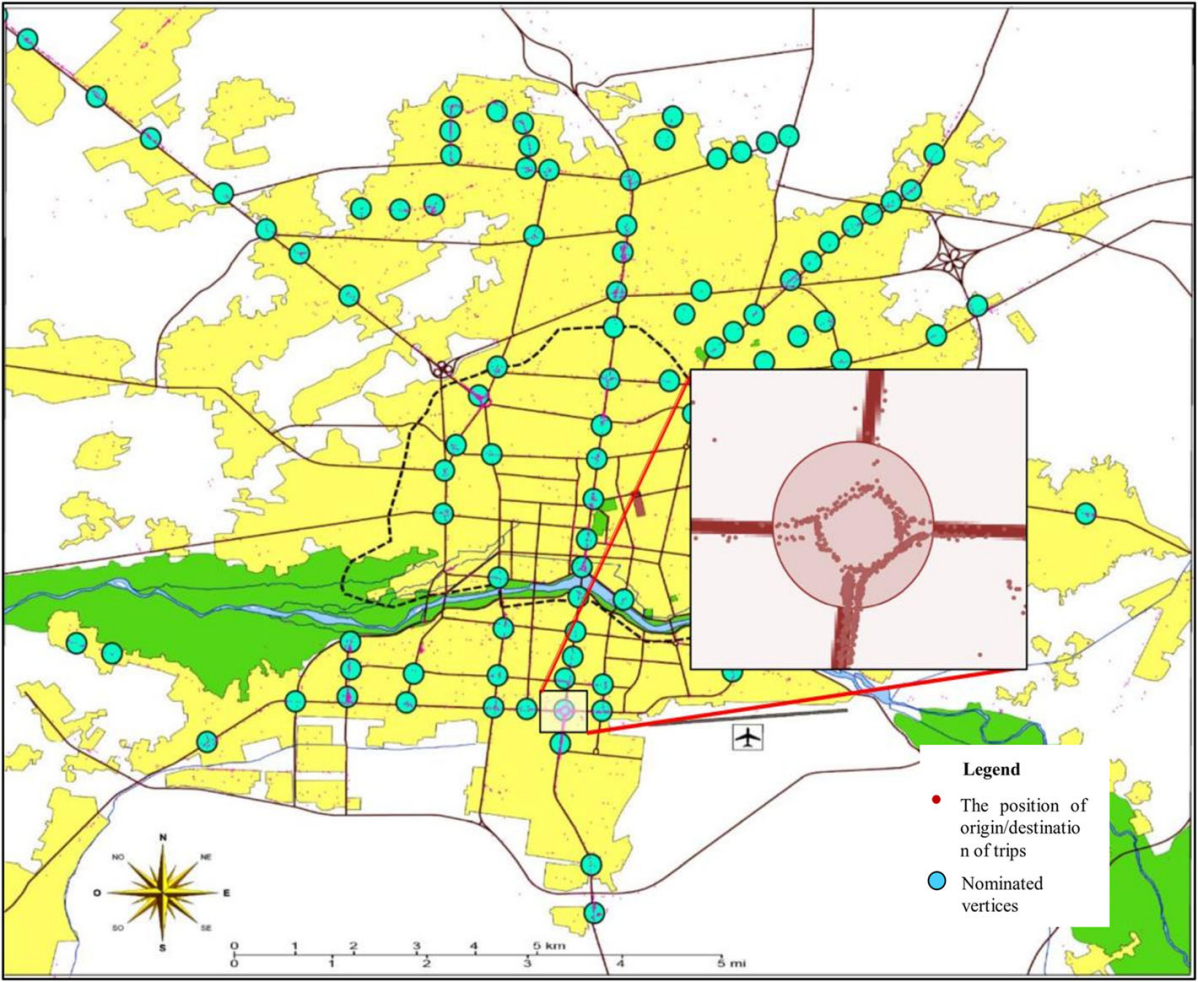
Spatial distribution of aggregated OD points

The taxi trips made on workdays were used to form the weighted directed network *G (N, E)* consisting of *n* nodes and *e* links. In order to form a network with tractable number of nodes, spatial aggregation was implemented on the origin and destination points. Nodes were assumed to be located at the intersections and major trip attracting areas of the city. Then every trip originated or destined within a circle of radius of 200 (m) around them, were aggregated. In other words, each node represents an area of trip generation and attraction with a radius of 200 m. Radius of these areas (200 m) was selected after considering the size of squares and intersections and relative position of nearby taxi stations. Figure 4 shows an example of aggregation of points within 200-m radius of a square.

**Fig. 5:**
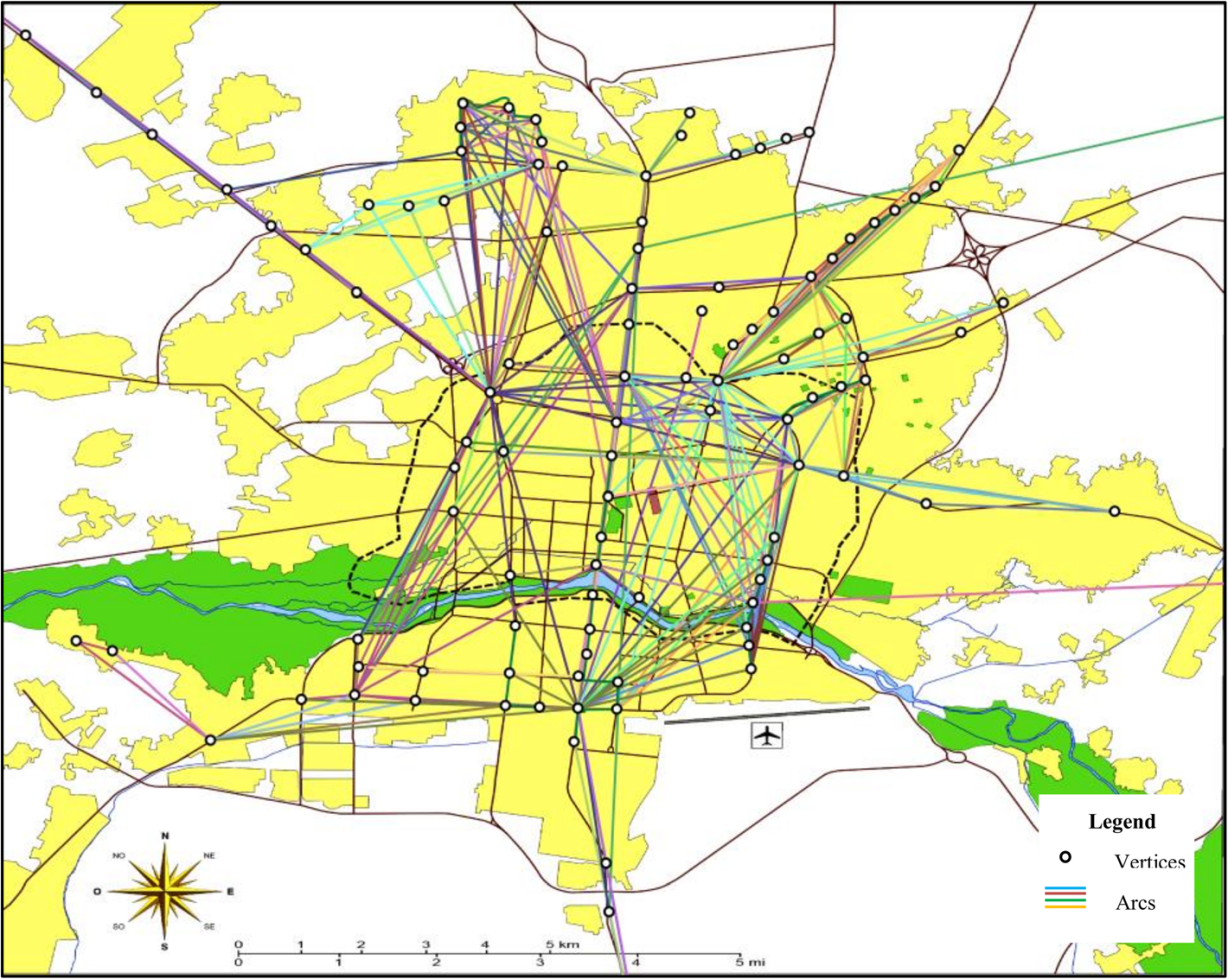
Vertices and arcs of Isfahan’s taxi network

The priority of each area for designation as a node was determined based on the number of trips generated and attracted to that area. After identifying the high priority areas on the Isfahan map, it was observed that almost 70% of all trips made in workdays pertained to 114 nodes. The links of the network represented direct trips among the nodes, and after aggregating the trips made between nodes, each link was assigned a weight equal to the total number of trips made on that particular route. Links were assumed to be undirected as the trips made by bike would be bidirectional. Next, the links with very low trip counts (less than 5 trips per day) were eliminated and the network of Isfahan’s taxi trips in workdays was developed with 114 nodes and 1112 links. Figure 5 shows a view of the network.

**Fig. 6:**
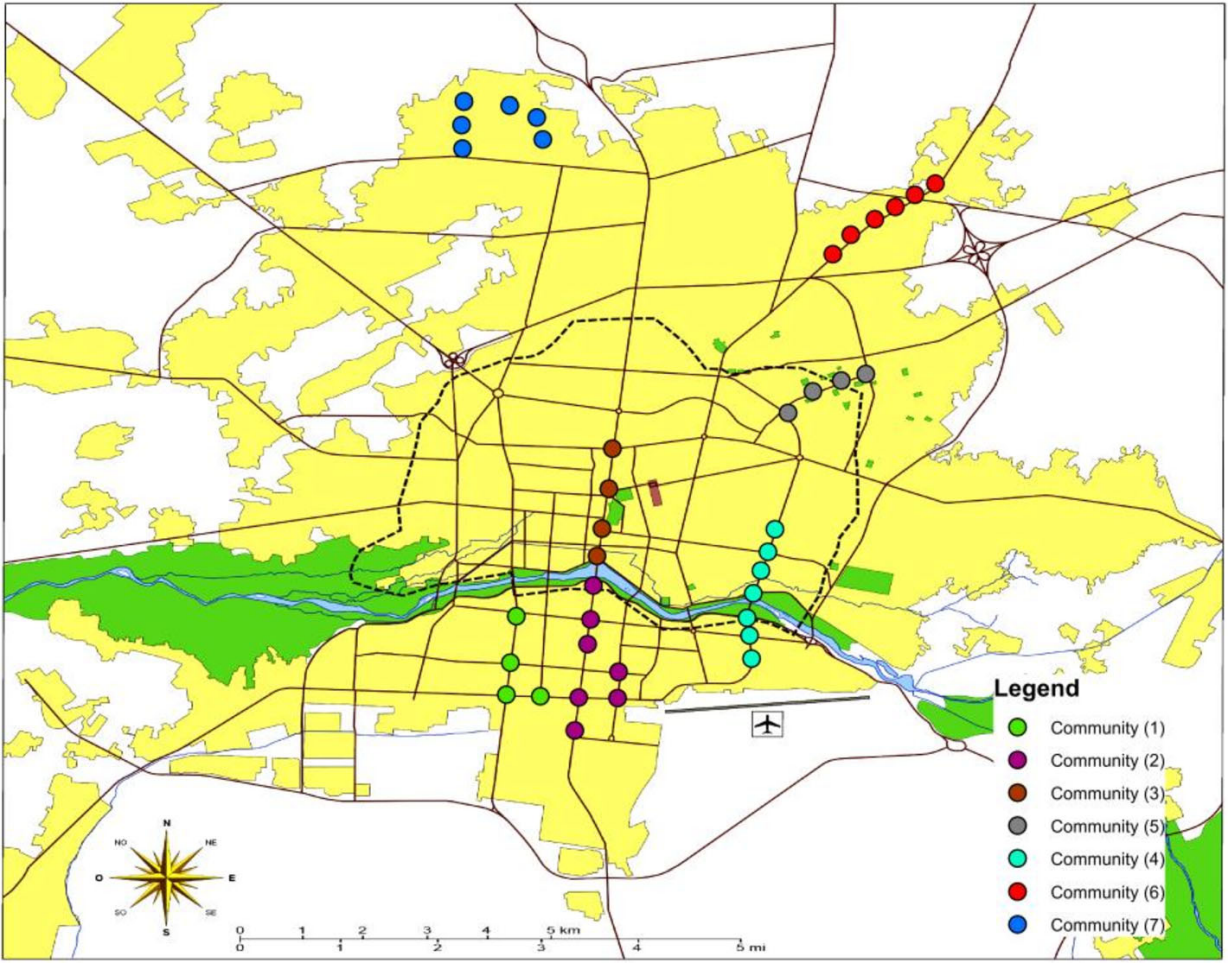
The key points in each community for constructing bike networks

The community detection algorithm detected seven clusters in the network. In each cluster, the node pairs with distance less than four kilometers were considered as key points for being located on future bike networks. The key points of clusters are illustrated in Fig. 6.

**Fig. 7:**
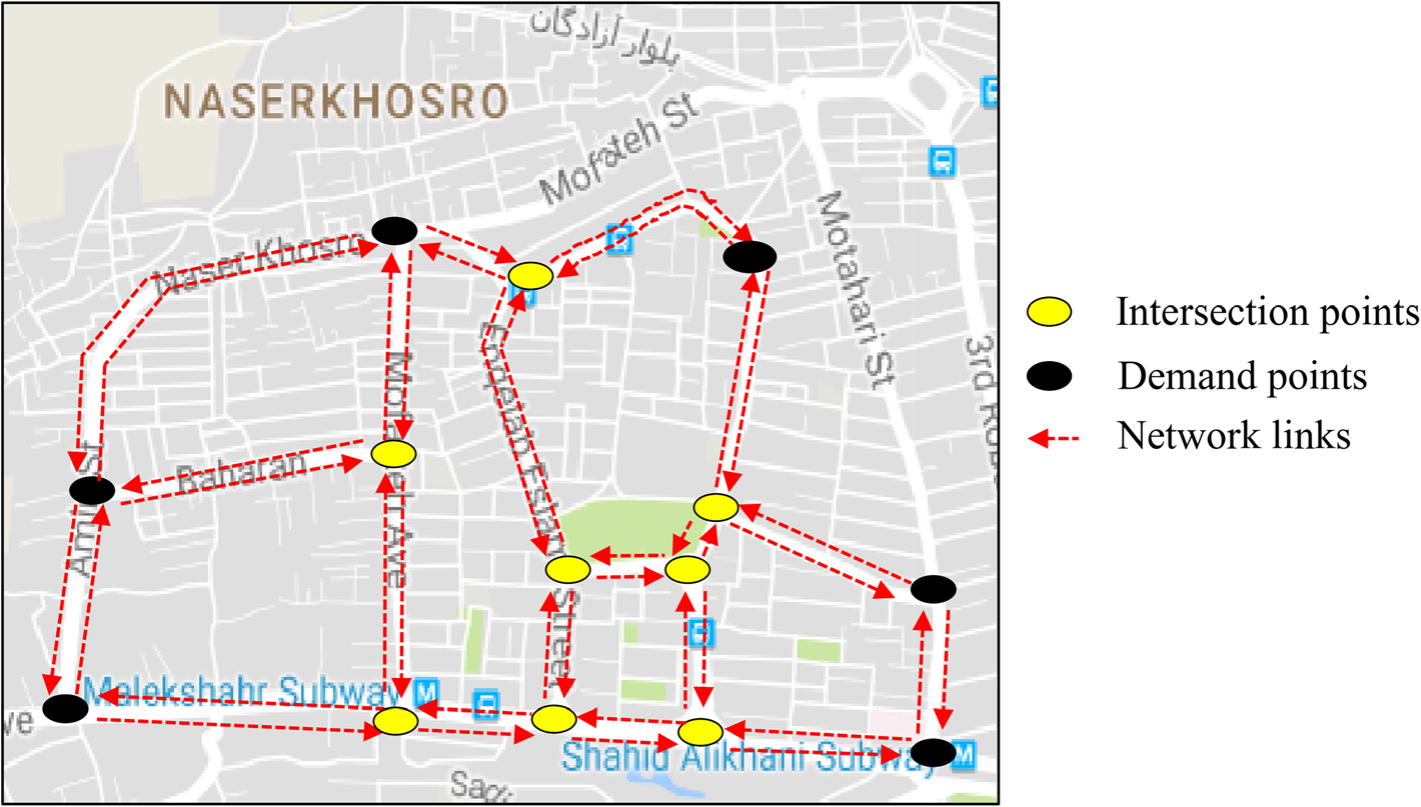
The proposed road network around key points of community 7

Once key points in each community were determined, some road networks around each key point were determined to extract the non-dominated bike networks. The key points in communities 3, 4, 5, and 6 are situated in a direct path. Therefore, applying the proposed model on them was not necessary and they have a non-dominated solution that connects the key points of the cluster to each other with a two-directed path. The bike networks for these communities are shown in Appendix. Figure 7 shows the proposed road network around the key points of community 7. Also, the proposed road networks around the key points of communities 1 and 2 are shown in Appendix. The proposed road network in community 7 consists of 14 nodes and 38 directed links. Among the 14 network nodes, the number of demand nodes is 6 and the eight remaining nodes belong to intersection nodes. The input parameters for running the model i.e. the length of network links and OD demand flows where extracted from Google Map and the data of taxi payment service, respectively.

The model was applied on the proposed road networks of communities 1, 2, and 7. Commercial software IBM ILOG CPLEX 12.6.1 was used for solving the bi-objective model in a device with Intel(R) Core(TM) i7 CPU @2.13 GHz and 6 Gbytes of RAM under the 64-bit Windows 7 operating system. In order to obtain an ideal solution for an objective in bi-objective programming models, its weight and the weight of the other objective were set equal to 1 and 0, respectively. In this situation the value of the other objective is equal to its nadir solution. Table 1 shows the obtained ideal and nadir solutions for all communities.

**Table 1:** The ideal and nadir solutions of bike networks of all communities

Community ID	User objective (*O*_1_) (bike-km)		Planners objective (*O*_2_) (km)	
	Ideal	Nadir	Ideal	Nadir
1	53.53	93.91	4.16	10.22
2	321.25	623.5	6.85	16.8
3	277.4	277.4	4.2	4.2
4	412.5	412.5	5.5	5.5
5	484.9	484.9	3.2	3.2
6	96.15	96.15	4.8	4.8
7	287.95	507.9	6.35	20.09

The results of finding the ideal and nadir solutions for communities 3, 4, 5, and 6 also confirm that these communities just have an optimal solution for both objectives. In order to find the non-dominated solutions for communities 1, 2, and 7, the weight of the first objective (*w*_1_) was increased from zero to one in increments of 0.1. It means that the weight of the second objective (*w*_2_) decreased from one to zero in increments of 0.1. Table 2 shows the characteristics of the non-dominated solutions for all communities. Some of the weighting systems yield same solutions which are illustrated in column 3 of Table 2.

**Table 2:** Characteristics of non-dominated networks for all communities

Community ID	$\sum \limits_{i\in K}\sum \limits_{j\in K}{h}_{ij}$	*w*_1_ (weight of the first objective)	network ID	User objective (*O*_1_) (bike-km)	Planner objective (*O*_2_) (km)
1	53	0	1	53.53	10.2
		All weights –{1,0}	2	54.35	4.3
		1	3	93.91	4.2
2	193	1	1	321.2	16.8
		0.9–0.8-0.7	2	328.7	8
		0.6–0.5-0.4-0.3	3	335.9	7.4
		0.2–0.1	4	374.4	7
		0	5	623.5	6.8
3	242	All weights	1	277.4	4.2
4	357	All weights	1	412.5	5.5
5	548	All weights	1	484.9	3.2
6	71	All weights	1	96.1	4.8
7	148	1	1	287.9	20.1
		0.9	2	288.2	15.3
		0.8–0.7	3	297.8	12.7
		0.6–0.5-0.4-0.3	4	336.3	8.3
		0.2–0.1	5	435.6	6.4
		0	6	507.9	6.3

**Fig. 8:**
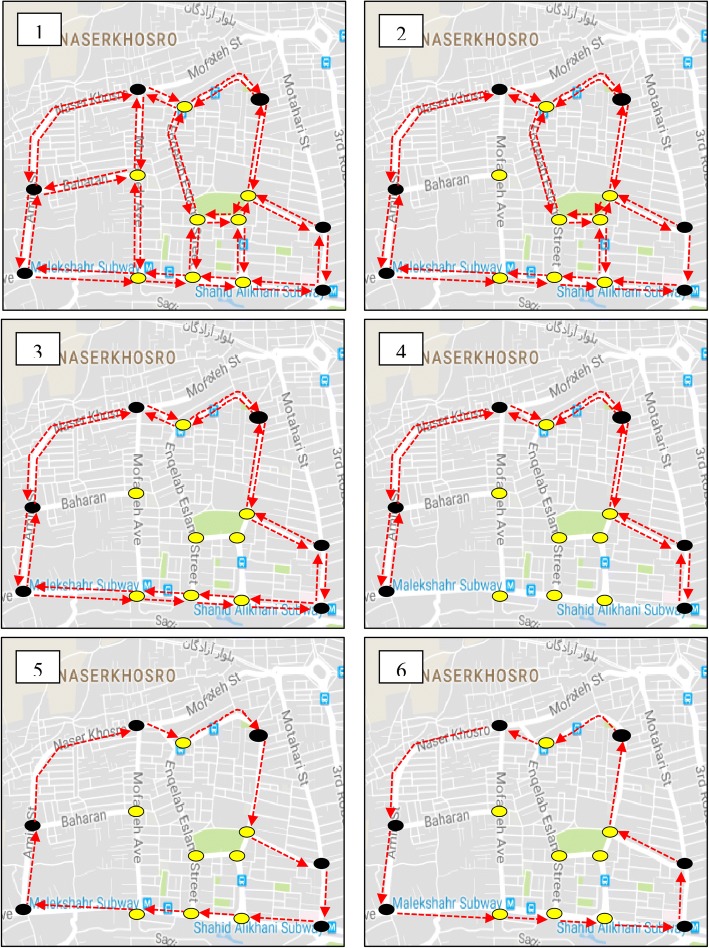
The proposed non-dominated bike networks for community 7

The model yielded 3, 5, and 6 non-dominated bike networks for communities 1, 2, and 7, respectively. A comparison between the objective values of non-dominated bike networks in each community shows that by making a little increase in users’ objective, it is possible to make a considerable improvement in planners’ objective and vice versa. For instance, consider the networks 1 and 2 in community 2. The value of the first objective in network 2 in comparison to network 1 is increased by 2.3%, while the second objective decreased by 52.3%. Therefore, with a little attention to the planners’ objective, one can decrease 50% of the total network length by another non-dominated bike network. As another example consider networks 4 and 5 in community 2. In this case with a little attention to users’ objective in network 4, the users’ objective is improved by 39.9%, while the planners’ objective is only increased by 2.1%. Figure 8 shows all non-dominated bike networks for community 7. The non-dominated bike networks for communities 1 and 2 are shown in Appendix.

**Fig. 9:**
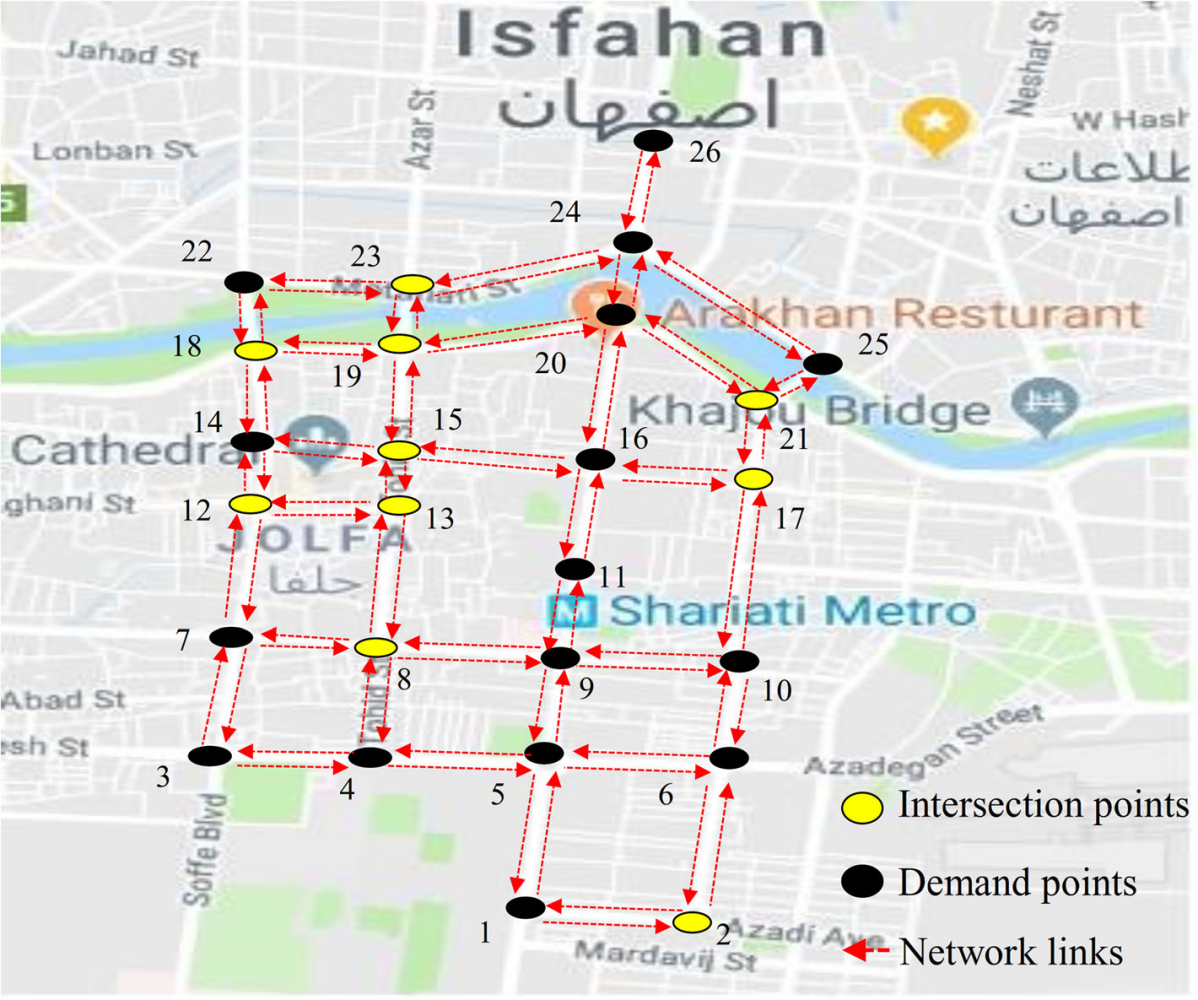
The road networks around the key points are situated in the picture area

In order to evaluate the quality of the proposed model, its performance in terms of speed, and trip coverage was compared to a random node selection approach. An extended covering area around communities 1 and 2 was selected and key points were randomly selected. Figure 9 shows the assumed area and its nominated nodes as well as the road facilities. The matrix of network demand is shown in Appendix: Table A.1.

The presented key points for community 2 in this area have a bigger captured OD flows than community 1. Therefore, the aim is comparing the values of model objectives for new and old (key points for community 2) sets. We believe that the total amount of OD flows between key points in new proposed sets affects the value of the model objectives and consequently disparages the process of comparison. For instance, if an alternative set was constructed with eliminating some key points of community 2, both model objectives would attain less values than prior and a dominating solution would be achieved. But this solution is derived from diminishing the total OD flows that is as important as two model objectives. In order to have a valid comparison, we can either consider the amount of captured OD flows as an additional objective or select some OD flows such that their sum is close to the amount of community 2.

We adopted the second approach into the proposed mathematical bi-objective bike network design by randomly selecting some key points from the network that their total OD flows have a maximum of 5% deviation of total OD flows in community 2 (193 ± 10 trips). We linked the Java software with commercial software IBM ILOG CPLEX 12.6.1 to choose a subset of network demand periodically and solved the model.

**Fig. 10:**
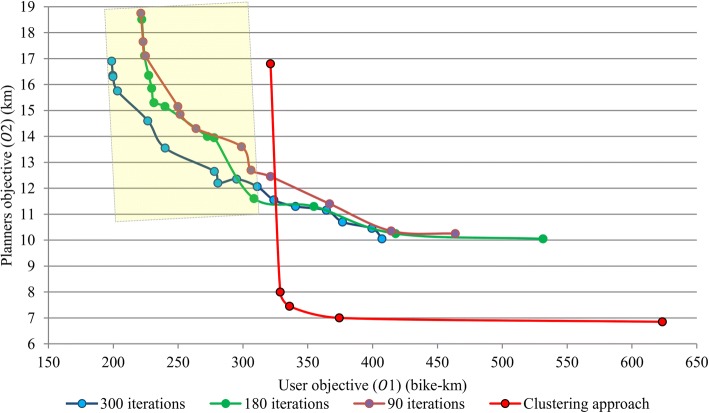
Demonstrating the quality of non-dominated bike networks based on presented approach

Figure 10 compares the quality of the obtained non-dominated solutions for 10, 30, and 50 iterations for each weighting system with the proposed non-dominated solutions of community 2 which were resulted by integrating network clustering with the optimization model. The total time for finding the communities of network and solving the mathematical model for each weighting system was 14 s. While, the recorded solution time without applying clustering approach for each weighting system were 301, 169, and 91 s with considering 300, 500, and 700 iterations, respectively. Therefore, the proposed approach is faster than random key points generation integrated with bike network design.

A first look at the non-dominated solutions of both approaches shows that the random key point selection approach could produce more optimal solutions from the standpoint of user objective than the presented clustering approach (solutions which are situated in yellow box). But this is caused by compromising the amount of total OD flows between the key points. Investigating the amount of total OD flows for solutions that are situated in the yellow box shows that they have a total OD flows up to 185 trips while the proposed model was applied on a network with 193 trips. Also, the clustering approach produced more optimal solutions from standpoint of planner objective even with reducing the total amount of OD flows in the network.

The proposed model belongs to the category of strategic problems for designing bike networks. In addition to identifying the links of a bike network, the number and position of bike stations is important. There are some other tactical problems that are concerned about locating the most suitable positon of bicycle stations and ensuring the adequate redistribution of bicycles. Adequate redistribution of bicycles increases the likelihood of stations servicing new passengers, increases the fleet productivity, and reduced the fleet size required to provide adequate service, which all in turn increase the demand and desirability of the whole program. These problems can be integrated with bike network design in future to propose a uniform network.

## Conclusion

A method for using the data collected by intelligent transportation system devices in planning urban infrastructure was proposed. This paper used the taxi trip data to suggest a number of bike networks for a city. The aim of this study was to provide a conceptual framework and a suitable approach for this purpose. The results of this paper can be further improved by repeating the work with a wider range of data, different community detection methods, engaging the attitude of residents, and trying different values for model parameters.

In this paper, the travel data of Isfahan’s taxis were used to extract the common origins and destinations of travels made by citizens. Then each set of relatively proximate points showing a high volume of exchange were classified as one community. Ultimately, authors proposed seven potential regions for setting up a bike network for Isfahan. In each community the vertices spaced less than 4 km from each other were considered as key points for designing a bike network. After identifying the key points in each community, with considering the road network types and their characteristics, in each community a network connecting the key points were proposed as bike network.

Next, a bi-objective optimization model was applied to each community to find the non-dominated bike networks. The first objective of the model minimized the total travel distance in network and was a users’ objective. The second objective minimized the total network length and was a planners’ objective.

The proposed method circumvents the need for collection of massive stated data on travelers’ trips and preferences. Since smart cards in bus and taxi are being rapidly embraced by cities, using their data does not incur extra charge. Although elegant, community detection is not a complicated and time consuming process. Therefore, the proposed method can be applied in almost every urban context.

## Figures and Tables

**Fig. 11:**
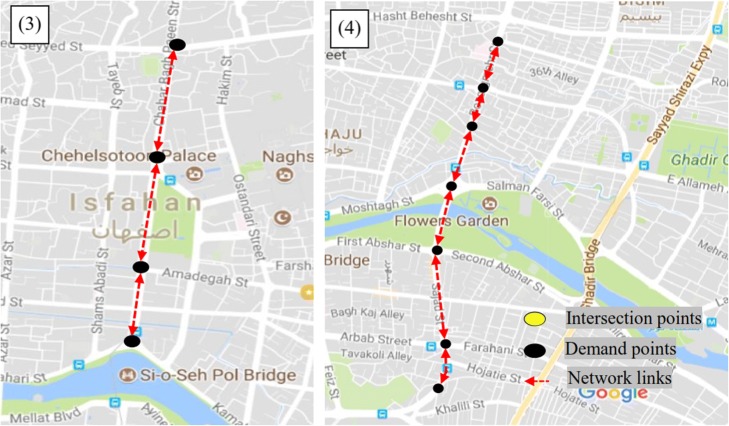
The best bike networks for communities 3 and 4

**Fig. 12:**
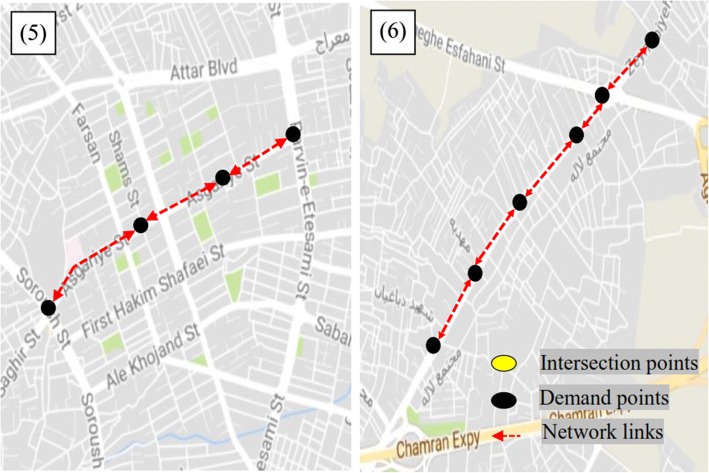
The best bike networks for communities 5 and 6

**Fig. 13:**
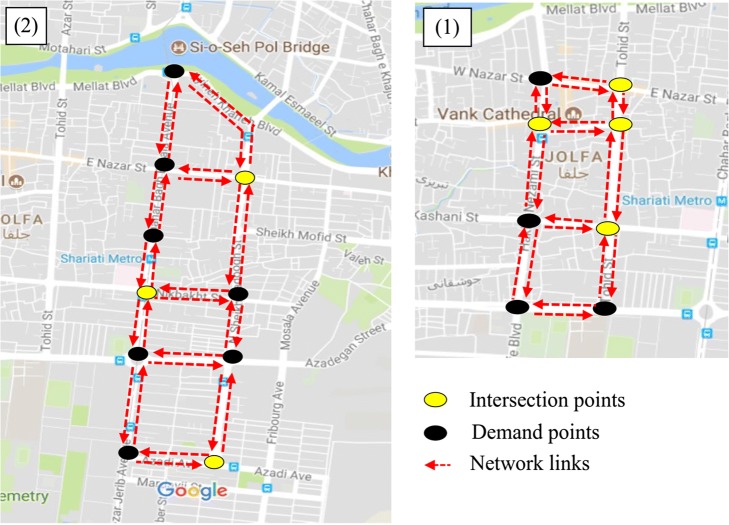
The proposed networks for community 1 and 2

**Fig. 14:**
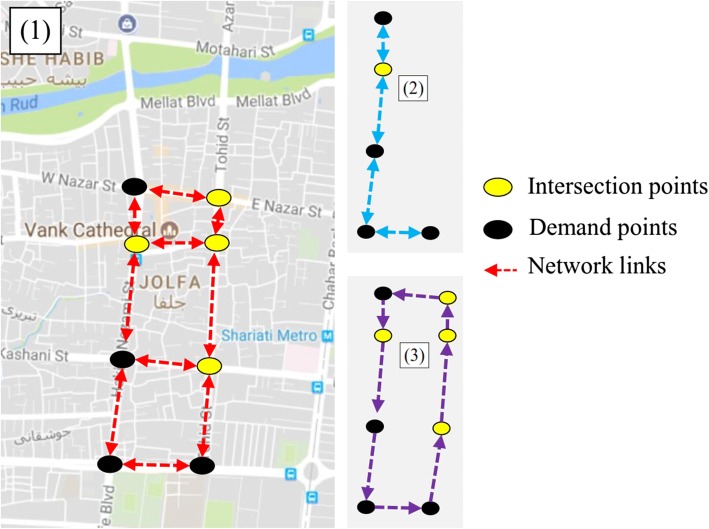
The proposed non-dominated bike networks for community 1

**Fig. 15:**
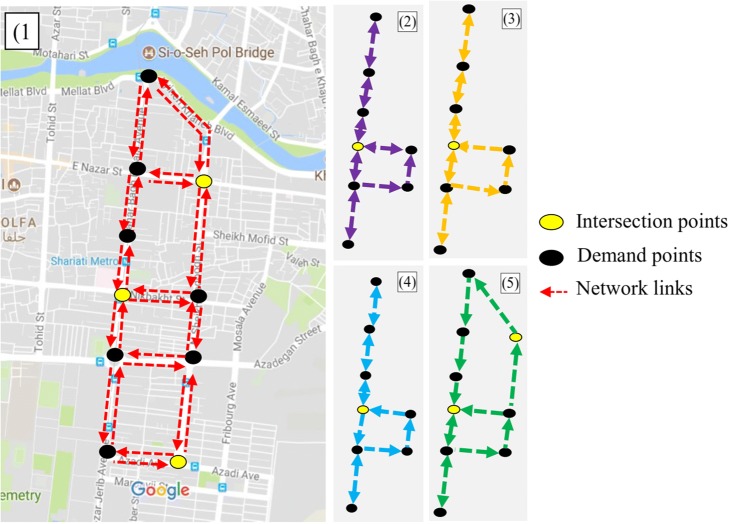
The proposed non-dominated bike networks for community 2

## Appendix

**Table 3:** OD flows for the network was considered around community 7

O/D	1	3	4	5	6	7	9	10	11	14	16	20	22	24	25	26
1				20								21		9		
3			10			3				5						
4		4				4				2						
5					6	6			14							
6								12				14				
7		6	4	4			3			3						
9						8		2						6		
10							1		8			18				
11	6										12	6				
14		6	1			5										
16												12				
20	12			14	10			8								
22														6		
24	11						8						11		6	40
25														3		
26														16		

## Abbreviation

OD: Origin Destination matrix/flow/pair
